# Optimization of sequence alignments according to the number of sequences vs. number of sites trade-off

**DOI:** 10.1186/s12859-015-0619-8

**Published:** 2015-06-09

**Authors:** Julien Y Dutheil, Emeric Figuet

**Affiliations:** 1 Department of Evolutionary Genetics, Max Planck Institute for Evolutionary Biology, August-Thienemann-Str. 2, Plön, 24306 Germany; 2 Institut des Sciences de l’Évolution - Montpellier, Place Eugène Bataillon – C.C. 065 –, Montpellier, 34095 France

**Keywords:** Sequence alignment, Comparative analysis prediction, Phylogeny, Sampling, Trade-off

## Abstract

**Background:**

Comparative analysis of homologous sequences enables the understanding of evolutionary patterns at the molecular level, unraveling the functional constraints that shaped the underlying genes. Bioinformatic pipelines for comparative sequence analysis typically include procedures for (i) alignment quality assessment and (ii) control of sequence redundancy. An additional, underassessed step is the control of the amount and distribution of missing data in sequence alignments. While the number of sequences available for a given gene typically increases with time, the site-specific coverage of each alignment position remains highly variable because of differences in sequencing and annotation quality, or simply because of biological variation. For any given alignment-based analysis, the selection of sequences thus defines a trade-off between the species representation and the quantity of sites with sufficient coverage to be included in the subsequent analyses.

**Results:**

We introduce an algorithm for the optimization of sequence alignments according to the number of sequences vs. number of sites trade-off. The algorithm uses a guide tree to compute scores for each bipartition of the alignment, allowing the recursive selection of sequence subsets with optimal combinations of sequence and site numbers. By applying our methods to two large data sets of several thousands of gene families, we show that significant site-specific coverage increases can be achieved while controlling for the species representation.

**Conclusions:**

The algorithm introduced in this work allows the control of the distribution of missing data in any sequence alignment by removing sequences to increase the number of sites with a defined minimum coverage. We advocate that our missing data optimization procedure in an important step which should be considered in comparative analysis pipelines, together with alignment quality assessment and control of sampled diversity. An open source C++ implementation is available at http://bioweb.me/physamp.

**Electronic supplementary material:**

The online version of this article (doi:10.1186/s12859-015-0619-8) contains supplementary material, which is available to authorized users.

## Background

By acting on the fate of mutations, natural selection shapes sequence variation at a given genomic locus. The analysis of sequence diversity therefore provides information on the underlying evolutionary forces, which in turn pinpoint the function of the encoded genes. For any gene of interest, obtaining and aligning homologous sequences from other individuals or species shed light on the evolutionary processes that generated the observed sequences. Sequence alignments are therefore the common entry point for many comparative methods, such as prediction of structure [1, 2], functional sites [3, 4], epistatic interactions [5, 6] and sites under positive selection [7].

The selection of homologous sequences is a critical initial step in comparative sequence analysis. For a given gene of interest, the number of available homologous sequences depends on the taxonomic distribution of the gene, which is itself a function of the age of the gene: ancient genes are shared by many taxonomic units, while recently evolved genes are more specific to a species or subset of species. To this *natural* distribution of homologues, one must add the actual sampling of species, which typically induces a bias toward model species and their relatives. This variability of gene representation in homologous sequence databases is also found at the intra-genic level. For a given family of homologous sequences, some positions are highly represented in the collection of sequences and present in most species. Other positions are found in only one or a few species. In the resulting sequence alignment, such positions would include gaps (‘-’) or unresolved characters (e.g., ‘N’, ‘X’ or ‘?’) depending on the cause of their absence. This variability of site-specific sequence coverage (further simply referred to as *coverage*) is a typical missing data problem. Depending on the downstream analyses, sites with insufficient coverage are not analyzable, or lead to inaccurate estimates.

Many homologous sequences are typically available as more and more organisms are sequenced. As a result, the number of sites with sufficient coverage, rather than the number of available sequences, is the limiting factor in comparative analysis applications. It is therefore beneficial for such applications to exclude sequences, provided that doing so increases the number of sites with sufficient data to be included in the analysis. While a few methods are available to filter badly aligned sequences [8] or redundant sequences [9], dedicated methods are needed to optimize sequence alignments for a given application based on site-specific coverage. We propose here a new algorithm whose specific task is to increase site coverage by sampling sequences from the alignment, here assumed to be correct. The algorithm uses a guide tree to compute scores for all partitions of the sequence data set and iteratively removes sequences responsible for low site-coverage, which allows optimization of the alignment size according to the number of sequences vs. number of sites trade-off.

## Methods

The method aims to increase the number of sites available for the subsequent analyses by sequentially removing the most costly sequences in terms of gaps or unresolved characters. It takes as an input a sequence alignment and a corresponding sequence clustering tree. The alignment optimization procedure is iterative, removing at each step the minimal group of sequences that adds the largest number of sites. The key component of the method is the computation of the putative gains of sites for all partitions (as defined by the input tree) as well as the corresponding costs in terms of sequences to be removed. Here we demonstrate that these computations can be efficiently achieved using dynamic programming and a two-traversal recursion on the tree.

### Computation of gains in sites for each group of sequences

**Fig. 1:**
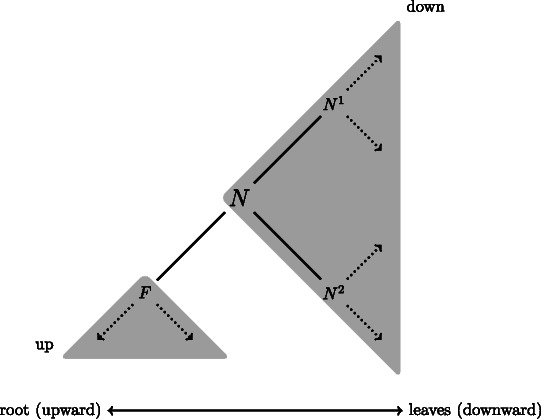
Notations. The tree is arbitrarily rooted and oriented. Any focal node *N* defines two partitions, noted *down* and *up* (the one containing the root of the tree). Neighbors of *N* are referred to as ’father’ node (*F*) and son nodes (*N* ^*k*^)

**Fig. 2:**
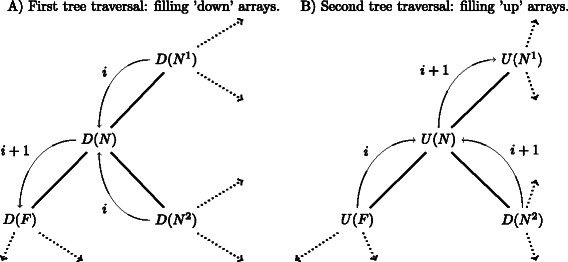
Tree traversals. Recursion orders for initializing *down* and *up* arrays. Notations are as introduced in Fig. 1. *i* and *i*+1 show operations performed during two subsequent iterations. **a** first, post-order tree traversal, initializing all *down* arrays from the leaves to the root. **b** second, pre-order tree traversal, initializing the *up* arrays from the root to the leaves





Given a sequence clustering tree arbitrarily rooted at an inner node, we define directions for tree traversals. For any node *N*, the neighbor node defining the subtree containing the root node is referred to as the ‘father’ node *F*, and all other neighbors (typically 2) are referred to as ‘son’ nodes *N* ^*k*^ (typically *N* ^1^ and *N* ^2^, see Fig. 1). For any node *N*, the branch connecting *N* to its father *F* partitions the sequences into two sets. We arbitrarily designate the *up* set of sequences as the one whose corresponding subtree contains the root of the tree and all others as the *down* set of sequences. For each node *N*, we store two score arrays *U*(*N*) and *D*(*N*), one for each set *up* and *down*, respectively. Each position in the array corresponds to one column in the alignment and specifies whether the column contains at least one gap in the corresponding sequence set. We proceed recursively to fill first the *D*(*N*) arrays, using Algorithm 1. For leaf nodes, the *D*(*N*)[*i*] array takes 0 if the underlying sequence has a gap in the alignment at column *i*; it takes 1 otherwise. The *D*(*N*) array of an inner node takes 1 if all son nodes have 1 at the corresponding position. All *D*(*N*) arrays at all nodes in the tree can then be computed recursively using a post-order tree traversal (Fig. 2a).

Once all *down* arrays have been filled, *up* arrays can be computed using another tree traversal. This second traversal computes scores for subtrees containing the root of the tree (Algorithm 2). For every node *N* with a father node *F*, the *U*(*N*) array is defined as the conjunction of the *U*(*F*) array and the *D*(*N*) arrays of all ‘brother nodes’, that is, all son nodes of *F* except node *N*. As *U*(*F*) needs to be computed before *U*(*N*), this second pass is a pre-order tree traversal (Fig. 2b).

After all *D*(*N*) and *U*(*N*) arrays have been computed, it is straightforward to compute for each node the number of sites for each corresponding partition, by summing all ‘1’ entries in each *up* and *down* array. The numbers of sequences for each partition can also easily be computed by recursion.

**Extension 1:** This method can be generalized to accommodate for sites with a given number of gaps or unresolved characters, defining a minimum coverage. In this extension, both *up* and *down* arrays contain a number of non-gap (or resolved) characters. During recursion (Algorithms 1 and 2), arrays are combined with a sum instead of the logical conjunction operator. The numbers of sites in each set of sequences is then computed by comparing for each site the proportion of gaps to the given threshold *t*∈[0,1]:

(1) $$ \sum_{i} \left[\frac{D(N)[i]}{n_{seq}(N)} \geq t\right]  $$

where $\sum _{i}$ is the sum over all columns in the alignment, [] are the Iverson brackets taking 1 if the enclosed condition is true and 0 otherwise, and *n*_*seq*_(*N*) is the number of sequences in the *down* subtree. Similar calculations apply for the *up* partitions. Such an extended algorithm requires more memory as counts typically require more space than boolean values. (In C++, the vector<bool> type uses less memory than the vector<int> type).

**Extension 2:** For some applications, one or several sequences in the input alignment are of particular interest, for instance because of availability of extra data (annotation, protein structure, *etc*.). It is therefore important (i) not to remove these sequences during the optimization process and (ii) to optimize the site coverage of these particular sequences. To accommodate for a set of reference sequences, the previous recursion can be applied on a guide tree where the reference sequences have been removed, with the extended equation 1:

(2) $$ \sum_{i} \left[\frac{D(N)[i] + R[i]}{n_{seq}(N) + n_{seq}(R)} \geq t\right]  $$

where *R*[*i*] is the number of characters at column *i* in the subalignment of reference sequences and *n*_*seq*_(*R*) is the number of reference sequences.

### Sorting partitions

The next step consists of ordering all partitions by relevance for the user. We implemented the following sorting scheme:

1. discard all sets of sequences where the number of sites is lower or equal to the current data set;
2. sort remaining sets of sequences by increasing number of sequences removed;
3. in case of a tie, sort the sets of sequences according to decreasing number of sites added;
4. in case of a tie, sort the sets of sequences according to decreasing total number of characters in the data matrix.

The decision regarding which set of sequences to keep (the current one or one of the proposed ones) is made either by the user or by a predefined criterion. In case a new partition of the data is selected, scores need to be recomputed.

### Update of partition scores

After one partition of the data set has been selected and the corresponding sequences removed, scores on the remaining tree have to be re-evaluated. Two cases have to be distinguished. In the first case, a *down* set of sequences is chosen and the tree is then restricted to the corresponding subtree matching the selected partition. All *down* scores are unchanged, but all *up* scores have to be recomputed using Algorithm 2. In the second case, an *up* set of sequences is favored and the subtree matching the complementary *down* set of sequences has to be removed from the current tree. All remaining subtrees need to have their *up* arrays updated using Algorithm 2. In addition, the *down* arrays for all nodes between the node defining the selected partition and the root have to be recomputed. The corresponding procedure is summarized as Algorithm 3.



### Termination

Several partitions can be selected sequentially to gradually increase the number of sites available by removing more sequences. The decision regarding when to stop the reduction loop can be left to the user (using an interactive implementation) or can be automated. A convenient automatic criterion is the maximum number of sequences to be removed. When a minimum final number of sequences is provided, the algorithm then finds the best combination of sequences to be removed that maximizes the number of sites. The program outputs the corresponding sampled alignment and tree.

### Sequence clustering tree

Any *a priori* hierarchical clustering of sequences, represented as a tree, can be used as an input to the method. The phylogeny of the underlying sequences, if known or inferable, is a natural choice. Yet for the purpose of optimizing the number of alignment columns without gaps, it is efficient to cluster the sequences according to their overlap in the alignment. For that purpose, we define the overlap distance between two sequences *S* ^1^ and *S* ^2^ from a multiple alignment with *n* columns as

(3) $$ {}d_{overlap}\left({S^{1}, S^{2}}\right) = n - \sum_{i=1}^{n} \left[{\left({{S^{1}_{i}} \neq\; 'NA'}\right) \wedge \left({{S^{2}_{i}} \neq\; 'NA'}\right)}\right].  $$

The sum represents the number of positions in the pairwise alignment for which both sequences do not have missing data (gap or generic character). All pairwise distances from the sequence alignment are computed using the overlap distance. The resulting pairwise distance matrix is then used as an input to standard hierarchical clustering procedures. Briefly, the clustering algorithm starts by defining as many clusters as there are sequences in the alignment, with one sequence each. It then proceeds iteratively, by grouping at each step the two clusters with the shortest distance in the matrix. The distance matrix is then reduced by one row and one column, and distances between the newly formed cluster and all other clusters in the matrix are recomputed. The method for updating distances between clusters of potentially more than one sequence defines the linkage type of the clustering procedure. In this work, we tested the following linkage type: complete, single, average, median, centroid and Ward [10]. Such clustering techniques, however, are not applicable for very large alignments as their complexity is typically *O*(*n* ^3^), where *n* is the number of sequences. For such large data sets (typically with more than 10,000 sequences), methods such as fasttree [11] are required to generate a guide tree.

### Example data sets

#### ORTHOMAM database

We used the ORTHOMAM database [12] version 8 [13], which gathers mammalian orthologous coding sequences based on ENSEMBL annotations. Nucleotide sequences for aligned orthologous gene coding sequences were retrieved with no filtering. Only alignments having at least 30 mammalian species out of the 40 available were kept, resulting in a total of 11,305 gene families. The phylogeny of the 40 species was downloaded from the ORTHOMAM website, and the corresponding phylogeny for each family was extracted by removing missing species. In addition, approximate maximum likelihood trees were inferred for each family using the Fasttree program [11]. For comparison, we applied our method on a random guide tree generated for each gene family using the rtree command from the R package *ape* [14]. Each species was searched in Pubmed to retrieve the number of publications with a title including its name (Additional file 1). This number of publications reflects the extent to which the species is studied and is typically high for model species such as *Homo sapiens* and *Mus musculus*. Correlation of the number of species with the frequency of sequence removal was performed using a Kendall correlation test as implemented in the R software. To correct for ties, input values were randomized 10 times using the jitter function. We report the minimum correlation value as well as the maximum p-value obtained over these 10 randomizations.

#### PFAM database

We retrieved all PFAM families with a number of sequences between 1,000 and 5,000 [15]. This selection comprises 2,785 protein families and includes species from bacteria, archaea and eukaryotes. The original PFAM alignments were used in all subsequent analyses. An approximate maximum likelihood phylogenetic tree was reconstructed for each family using the Fasttree program [11] as well as a random tree using the rtree command from the R package *ape* [14]. As opposed to the ORTHOMAM benchmark data set, families in the PFAM benchmark might contain paralogous sequences.

### Software availability

The algorithm and its extensions described in this work were implemented in C++ using the Bio++ libraries version 2.2.0 [16]. The resulting program called *bppalnoptim* is available under the General Public License version 3.0 (GPL3) at http://bioweb.me/physamp.

## Results

We applied the new optimization algorithm to two contrasting data sets to cover a wide range of potential applications. First, we analyzed 11,305 mammalian gene families from the ORTHOMAM database [13]. Each family contained one orthologous sequence for at least 30 species of mammals. Second, we studied 2,785 protein families, each having between 1,000 and 5,000 sequences, from the PFAM database. In both cases, we aimed to maximize the number of sites with a given minimum coverage: we allowed a maximum of 5 % and 30 % sequence gaps and unresolved characters for ORTHOMAM and PFAM, respectively. The two data sets have contrasting dimensions: the ORTHOMAM data set contains a large number of sites but few sequences, while the PFAM families contain a large number of sequences but typically very few sites.

### Visualizing the number of sequences vs. number of sites trade-off

**Fig. 3:**
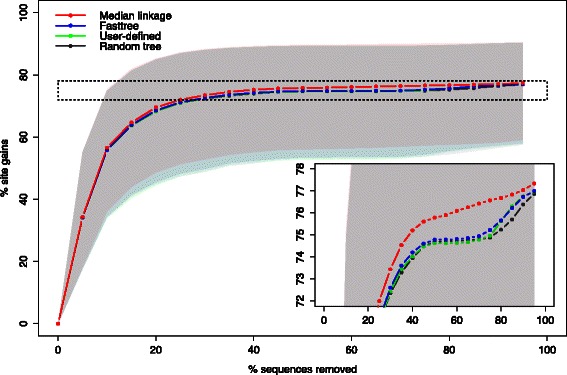
ORTHOMAM trade-off curves. Proportion of site gains (final number of sites - initial number of sites, divided by the final number of sites) as a function of the proportion of sequences removed. Points show the median over the 11,305 gene families, and shaded areas show the first (25 %) and third (75 %) quartiles. Three methods are superimposed: hierarchical clustering of sequences using the overlap distance and median linkage, the fasttree maximum likelihood tree reconstruction, and a fixed tree representing the known phylogeny of species

We applied our algorithm on each family independently and compared different methods to generate the guide tree. To assess the efficiency of each method, we visualized the optimization trade-off by plotting for each family the number of sites gained (as a proportion of the maximum number of sites) as a function of the number of sequences removed (as a proportion of the initial number of sequences). Figure 3 shows the optimization trade-off curves for the ORTHOMAM data set, with the curve and corresponding shaded area showing the median, first and third quartile of the results for the 11,305 families. The curves revealed the existence of a maximum number of sites reached by all methods when approximately 30 % of the sequences were removed. The average maximum gain in the number of sites was above 75 %, which corresponds to a tripling of the number of sites available for the analysis. While we noticed a slightly better performance when the guide tree was constructed using the overlap distance, a random tree achieved similar results on average for this data set. Furthermore, we did not find any difference between clustering methods based on the overlap distance (Additional file 2: Figure S1).

**Fig. 4:**
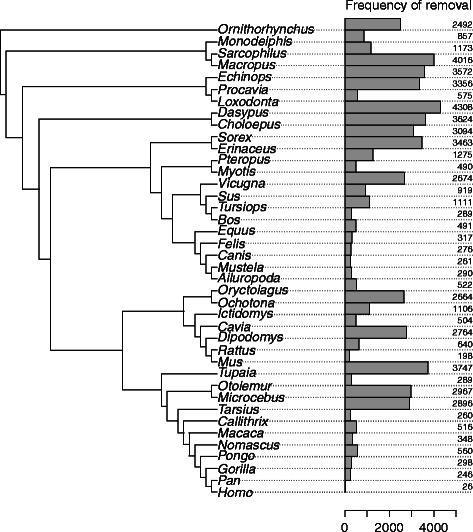
Frequencies of species removal. Phylogeny of the 40 species in the ORTHOMAM data set, with their corresponding frequency of removal. Bars and numbers indicate the number of families where the given species was present in the original alignment but removed after optimization

We further investigated which sequences were removed for each family, using Fasttree to generate guide trees. The stopping condition for the algorithm was to keep a minimum of 80 % of the original sequences. We showed that all species were excluded in at least one family (Fig. 4). The frequency of removal reflects the quality of genome annotation: there is a significant negative correlation between the frequency of removal of species and the number of articles in Pubmed containing the species name in their title (Kendall’s tau = -0.264, p-value = 0.0185). Model organisms that are studied more and therefore benefit from manual annotation of genes were less frequently removed than species for which gene prediction relies mostly on *de novo* and homology-based predictions.

**Fig. 5:**
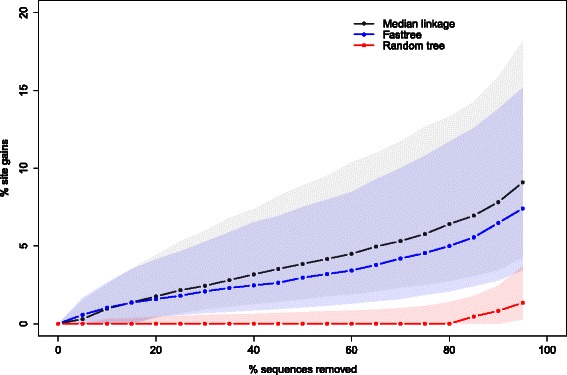
PFAM trade-off curves. Same representation as used in Fig. 3 for the 2,785 families of the PFAM data set

The trade-off curves obtained from the PFAM data set exhibited a different shape, with the site gain linearly increasing as more sequences were removed (Fig. 5). As for the ORTHOMAM benchmark, the overlap distance clustering led to a slightly better performance than the fasttree method, while no difference was observed on average between clustering methods (Additional file 3: Figure S2). As opposed to the ORTHOMAM benchmark, the PFAM data set demonstrated the effect of the choice of a relevant guide tree for data sets with greater sequence divergences: the performance of the algorithm dropped to nearly zero site gain when a random tree was used as a guide (Fig. 5).

**Fig. 6:**
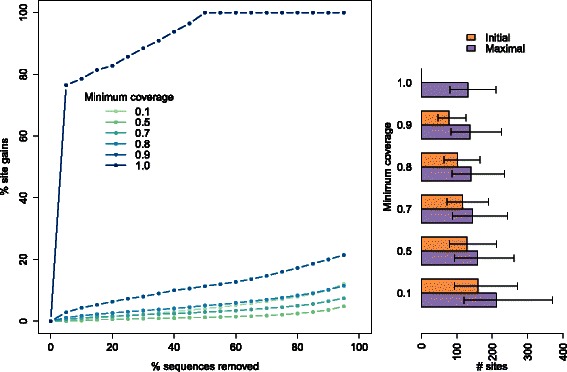
Trade-off curve and minimum coverage. Trade-off curves using the fasttree guide tree as in Fig. 5, plotted for different minimum coverage values required for sites to be included in the analysis. A minimum coverage of 1.0 implies that only complete columns with no gap and no unresolved characters are included. Bars on the right show the number of sites with the required minimum coverage for each family (Initial), as well as the maximum number of such sites reachable by optimization (Maximal). Bars show the median numbers over all families, intervals represent the first (25 %) and third (75 %) quartiles

As the PFAM alignments were much larger and had a higher underlying sequence diversity, we assessed the effect of the minimum coverage set for defining a site as included in the analysis. We used the output of fasttree to generate a guide tree. The corresponding trade-off curves showed that the relative site gain increased with the minimum coverage, but this effect was largely due to the number of sites initially fitting the given criterion being very low (Fig. 6 and Additional file 4: Figure S3). As much as 85 % (2,360 out of 2,785) of the families had no complete site (defined as having a coverage of 1.0, that is, with no gap and no unresolved character). Globally, our method was successful in increasing the number of sites available for data analysis in PFAM families while controlling for the number of sequences filtered.

## Discussion

### Applications of the optimization algorithm

For many gene families, the number of sequences available for comparative analysis pipelines (e.g. evolutionary rate estimation [17], positive selection detection [7] and coevolution analysis [6]) is typically large. While extraneous sequences will only marginally contribute to the extracted signal, they will significantly impair most methods by increasing the execution time and memory usage. It is therefore common practice to discard some sequences at early preprocessing stages, and it is relevant to do so in a way that will minimize the proportion of missing data. Our method is therefore useful when (i) the size of the data set must be reduced for computational efficiency and/or (ii) missing data are expected to introduce noise or bias in downstream analyses.

Missing data are present in sequence alignments as unresolved characters (‘N’, ‘X’ or ‘?’), and it is also common practice for some applications to recode gaps (‘-’) as missing data. The units of alignment-based analysis are sites (i.e. alignment columns), and sites with insufficient coverage (that is, with too much missing data) are usually discarded in downstream analyses as they may lead to unreliable estimates or introduce noise. As the desired minimum site-specific coverage depends on the particular downstream analysis, it is an input parameter of our method. We have proposed an algorithm that increases the number of sites matching the required threshold by selectively removing sequences with missing data. For example, coevolution detection methods typically work better with no missing data because two sites with an unresolved character or a gap in the same species will show an artificially high correlation. For such methods, the desired input threshold is therefore typically conservative, allowing little or no missing data at analyzed positions. Conversely, rate estimation methods are more robust in the presence of unresolved characters, and a more permissive threshold can be used.

**Fig. 7:**
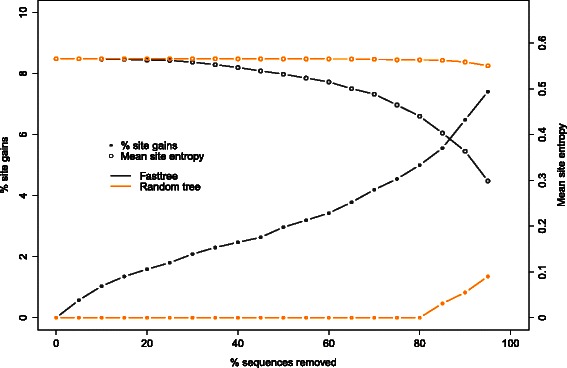
Trade-off curve and average site entropy. Trade-off curves using the fasttree guide tree together with a random tree as in Fig. 5, plotted along with the corresponding mean site entropy

It is noteworthy that another criterion for removing sequences from an alignment while maintaining most of its biological signal is to remove identical or highly similar sequences, for instance using the CD-HIT software [9]. Such similarity-based approaches are complementary to the missing data reduction method described here because they exploit distinct properties of the data set. Because sequence similarity measures are affected by the occurrence of missing data and because our algorithm is independent of the similarity of input sequences, we recommend using our method prior to similarity reduction filters such as CD-HIT. Because similar sequences tend to have missing data at similar positions (gaps in particular), the sequential removal of sequences tend to remove more distantly related sequences and reduce the global diversity of the input alignment. To illustrate this aspect, we computed the Shannon entropy for each site and computed the average over all sites as an estimate of the sequence diversity in an alignment. Using the PFAM data set as an example, we plotted this measure of diversity along the trade-off curve (Fig. 7). We report a decrease of diversity as more sequences are removed, and this decrease is faster for large sequence removal (more than 60 % sequences removed). Conversely, the random removal of sequences keeps diversity constant but does not improve coverage. The relative amount of sequence diversity in the optimized data set can be used as a stopping condition for the optimization algorithm.

### Importance of guide tree

We have shown that for relatively similar sequences, the order in which sequences are compared, as specified by the guiding tree, has virtually no impact on the resulting selection. For more dissimilar sequences, however, better results are achieved when sequences are clustered according to their respective overlap, as measured by the overlap distance introduced in this work. As such, a clustering procedure can be computationally expensive; an optimal approach consists of using a fast phylogenetic tree reconstruction method such as Fasttree to generate a guide tree without compromising the quality of the results.

### Termination criterion

The algorithm introduced in this work proposes a criterion to recursively remove sequences that are costly in terms of site coverage. Distinct cases should be distinguished when establishing the criterion for preventing the removal of additional sequences. A straightforward case is when no more improvement can be achieved, either because all sites match the requested minimal coverage or because the removal of any additional (group of) sequence(s) will not lead to any improvement. Such a situation arises when missing data are uniformly distributed among sites: removing a sequence might gain new sites but will also lead to the loss of others so that the net site gain is null. In some other cases, as illustrated by our ORTHOMAM example data set, the site gain reaches a plateau, meaning that an increasing number of sequences have to be removed to gain additional sites (Fig. 3). Such curves resemble rarefaction curves [18]. In such cases, it is possible to define a cost parameter as a maximum number of sequences to be removed per additional site gained. The optimization of the data set will then proceed until the cost of obtaining additional sites exceeds the given threshold. Other data sets, however, display a linear trade-off curve (PFAM example data set, Fig. 5). For such data sets, a fixed cost parameter will lead to the removal of all or no sequences, if the slope of the curve is lower or higher, respectively, than the given parameter. In such a case, the stopping condition of the algorithm is based on the desired number of sites or sequences in the filtered data set. Our implementation of the algorithm allows these two stopping conditions, which can be combined. We have also implemented a diagnostic mode, which allows drawing the trade-off curve of the data, as in Figs. 3 and 5. Plotting the trade-off curve is helpful to visualize the distribution of missing data in a given data set, to decide which criteria to use for its subsequent optimization.

### Alignment optimization vs. alignment quality assessment

Errors in sequence alignment reconstruction have been the subject of several studies [19-22]. Such errors propagate at downstream stages of the analysis, inflating the false discovery rate. Several approaches have therefore been proposed to specifically address this issue [8, 23, 24]. These methods compute a quality score for each alignment position and discard the positions that are considered too uncertain. While site-filtering procedures are necessary to increase prediction accuracy, they come at the cost of a loss of statistical power due to the shrinkage of sites available for further analysis. This removal of ambiguously aligned sites further reduces the number of sites available for downstream analyses. The most advanced alignment filtering procedures such as TrimAl [8] and Guidance [24] also permit filtering the alignment sequence-wise, which increases the global quality of the alignment by removing dubiously aligned sequences, therefore increasing site-specific quality scores. Such methods are designed for increasing alignment quality and should therefore be used to complement the optimization algorithm described in this work. An interesting application is the recursive use of our optimization algorithm and alignment methods. By removing sequences that are costly in terms of missing data, the alignment of the remaining sequences could potentially be improved.

## Conclusions

Typical bioinformatics pipelines include upstream processing steps to filter alignment errors and control for sequence redundancy. We have proposed here an additional step to optimize data sets based on their missing data content and have introduced an efficient algorithm for that purpose. Such optimization is achieved by removing costly sequences from the alignment to increase the number of alignment columns with minimal sequence coverage. The proposed algorithm uses a guide tree, which can be constructed from the input alignment using clustering techniques or provided by the user, for instance, as a phylogenetic tree. Using two complementary benchmark data sets of several thousands alignments each, we have demonstrated the potential broad usage of this new algorithm. We posit that as families of homologous sequences further increase in size, optimal sampling of sequence alignments will become a necessary complement to alignment quality check procedures to maximize the power and accuracy of comparative analysis-based predictions.

## Additional files

Additional file 1
**Figure S1.** Trade-off curves for the ORTHOMAM benchmark data set. Each panel represents a distinct procedure for generating the guide tree. The solid line shows the median over all 11,305 families; the shaded area represents the first (25 %) and third (75 %) quartiles.

Additional file 2
**Figure S3.** Trade-off curves for the PFAM benchmark data set, using the output of Fasttree as a guide tree. Each panel represents a distinct minimum coverage for a site to be included in the analysis. The solid line shows the median over all 2,785 families; the shaded area represents the first (25 %) and third (75 %) quartiles.

Additional file 3
**Figure S2.** Trade-off curves for the PFAM benchmark data set. Each panel represents a distinct procedure for generating the guide tree. The solid line shows the median over all 2,785 families; the shaded area represents the first (25 %) and third (75 %) quartiles.

Additional file 4
**Raw data for number of citations per ORTHOMAM species.** A coma-separated value (CSV) text file containing the frequency of removal of each ORTHOMAM species, together with the number of publications containing the species name in their title. The #-commented lines at the start of the CSV file contain R code to reproduce the Kendall’s correlation test reported in this work.
