# Supplementary material for: Optimization of sequence alignments according to the number of sequences vs. number of sites trade-off
Source: BMC Bioinformatics. 2015 Jun 9;16:190. doi: 10.1186/s12859-015-0619-8 (PMC4459672; doi:10.1186/s12859-015-0619-8)
Supplement: Additional file 1 — Figure S1. Trade-off curves for the ORTHOMAM benchmark data set. Each panel represents a distinct procedure for generating the guide tree. The solid line shows the median over all 11,305 families; the shaded area represents the first (25 %) and third (75 %) quartiles. [file 12859_2015_619_MOESM1_ESM.pdf]

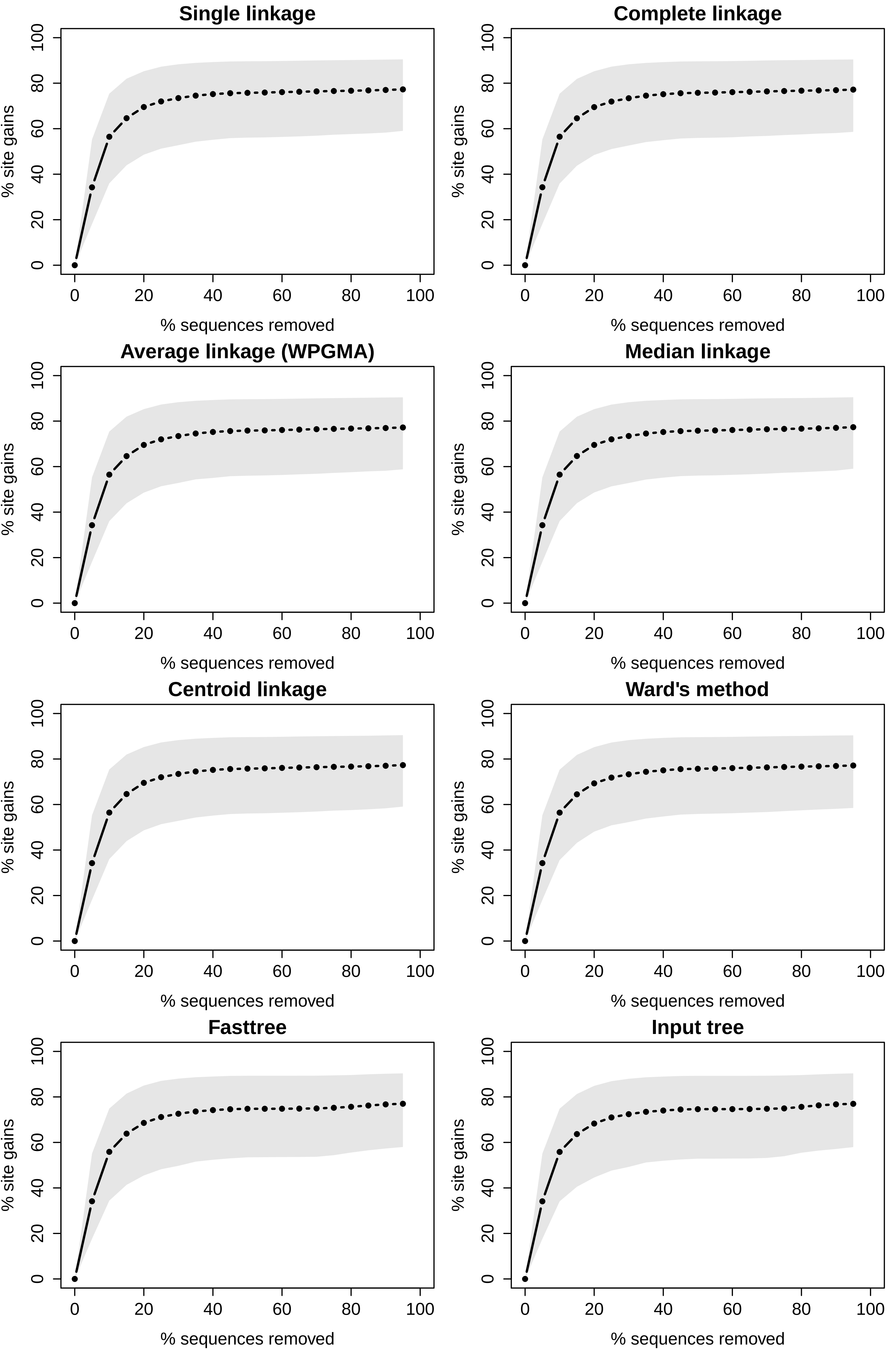
Single linkage
Complete linkage
Average linkage (WPGMA)
Median linkage
Centroid linkage
Ward's method
Fasttree
Input tree
% site gains
% sequences removed
0
20
40
60
80
100
