# Supplementary material for: Optimization of sequence alignments according to the number of sequences vs. number of sites trade-off
Source: BMC Bioinformatics. 2015 Jun 9;16:190. doi: 10.1186/s12859-015-0619-8 (PMC4459672; doi:10.1186/s12859-015-0619-8)
Supplement: Additional file 2 — Figure S3. Trade-off curves for the PFAM benchmark data set, using the output of Fasttree as a guide tree. Each panel represents a distinct minimum coverage for a site to be included in the analysis. The solid line shows the median over all 2,785 families; the shaded area represents the first (25 %) and third (75 %) quartiles. [file 12859_2015_619_MOESM2_ESM.pdf]

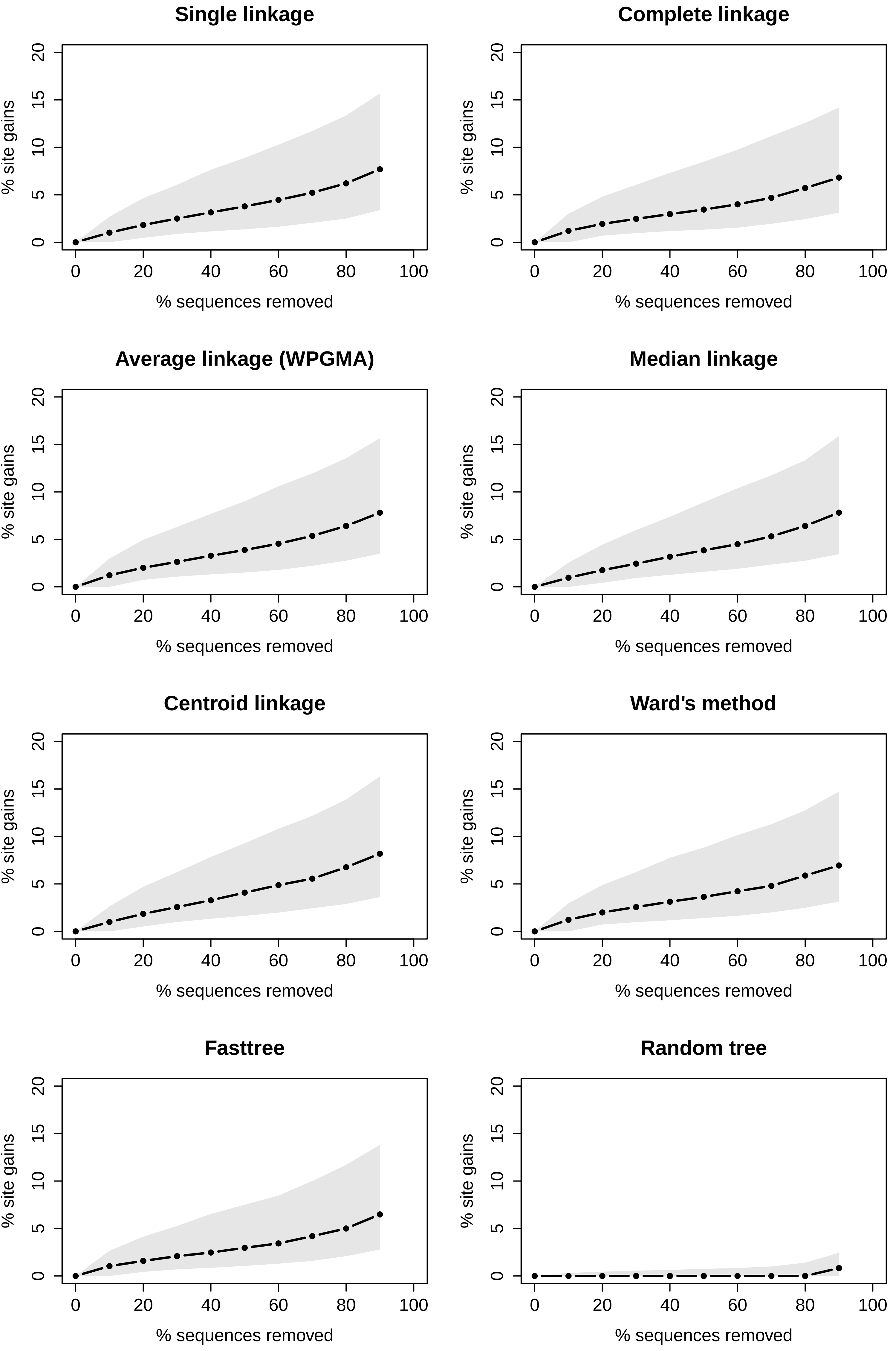

Single linkage
Complete linkage
Average linkage (WPGMA)
Median linkage
Centroid linkage
Ward's method
Fasttree
Random tree
% site gains
% sequences removed
0
5
10
15
20
0
20
40
60
80
100
