# Supplementary figures and images for: Optimization of sequence alignments according to the number of sequences vs. number of sites trade-off
Source: BMC Bioinformatics. 2015 Jun 9;16:190. doi: 10.1186/s12859-015-0619-8 (PMC4459672; doi:10.1186/s12859-015-0619-8)

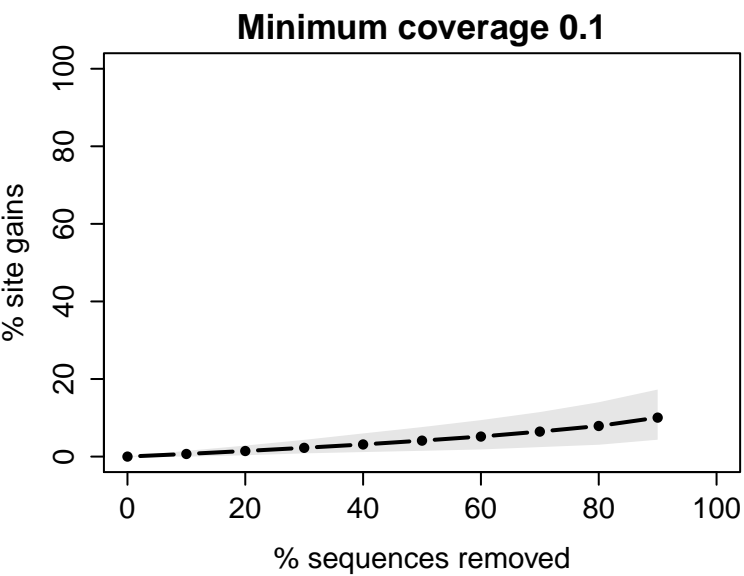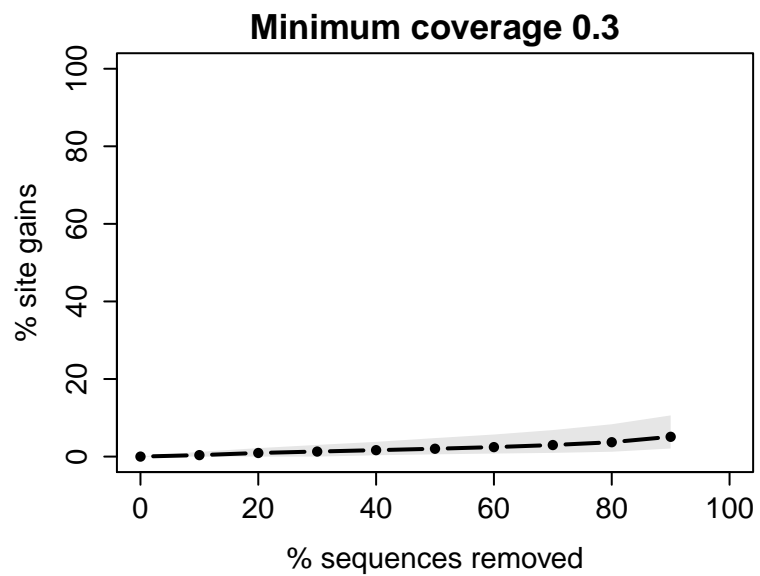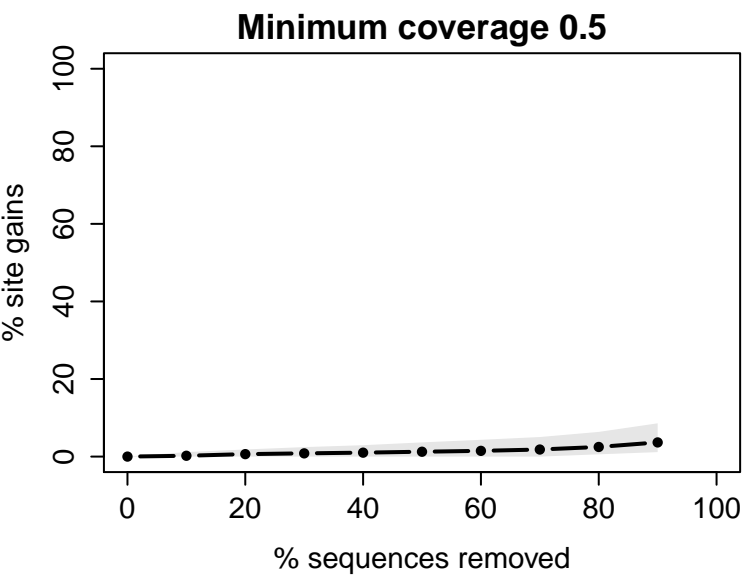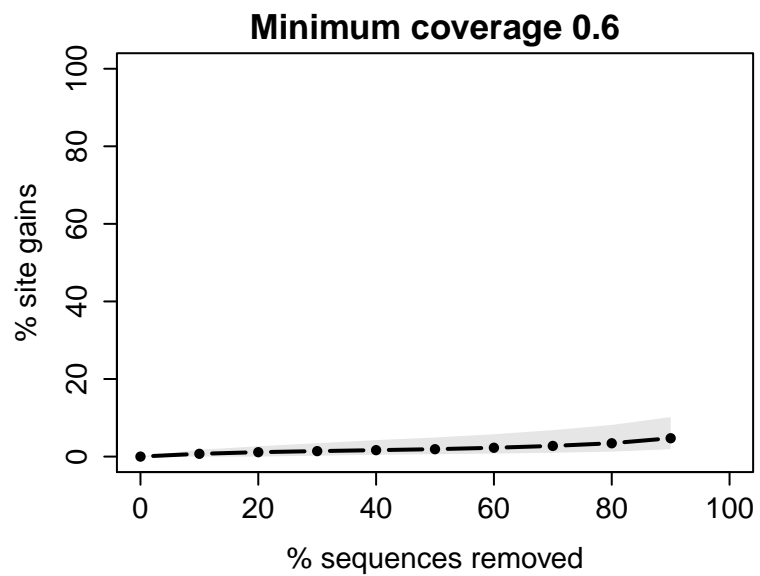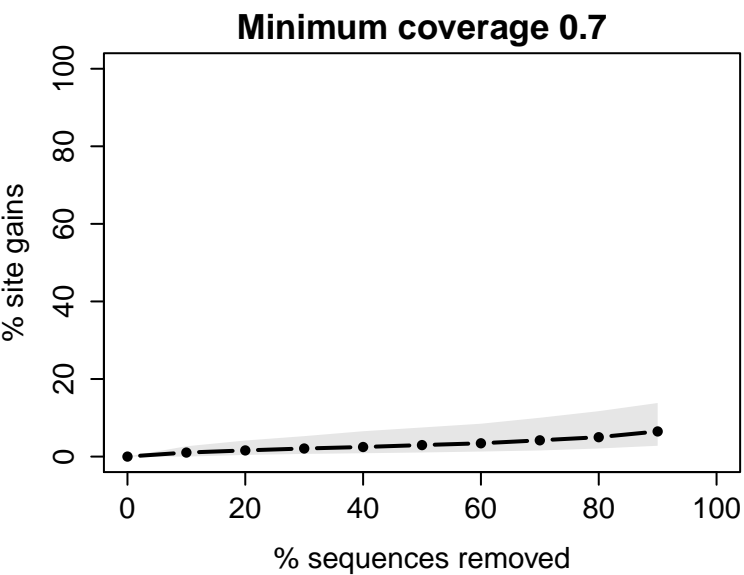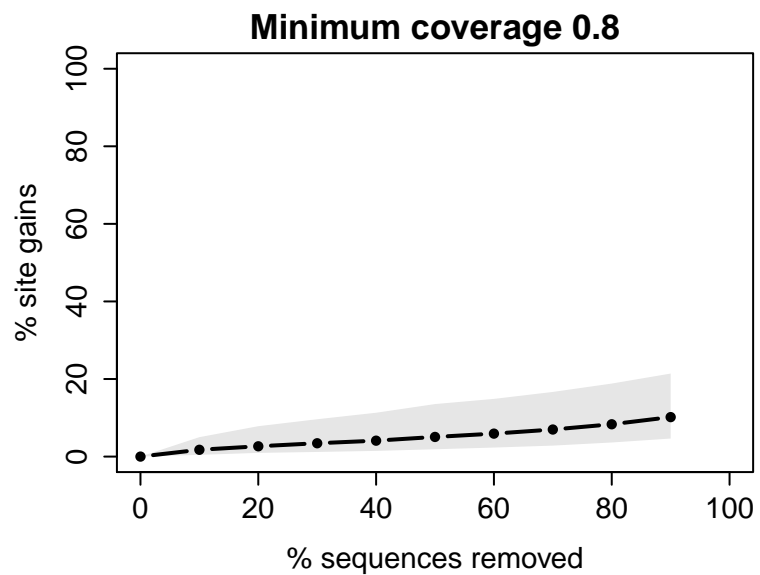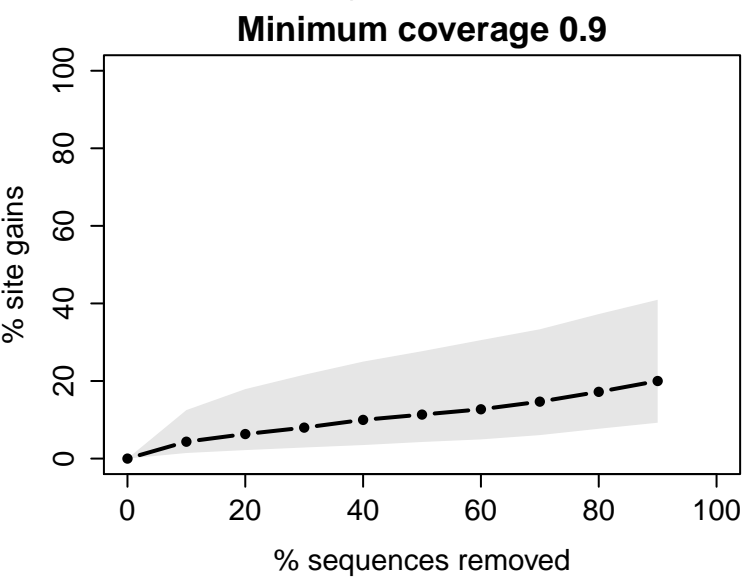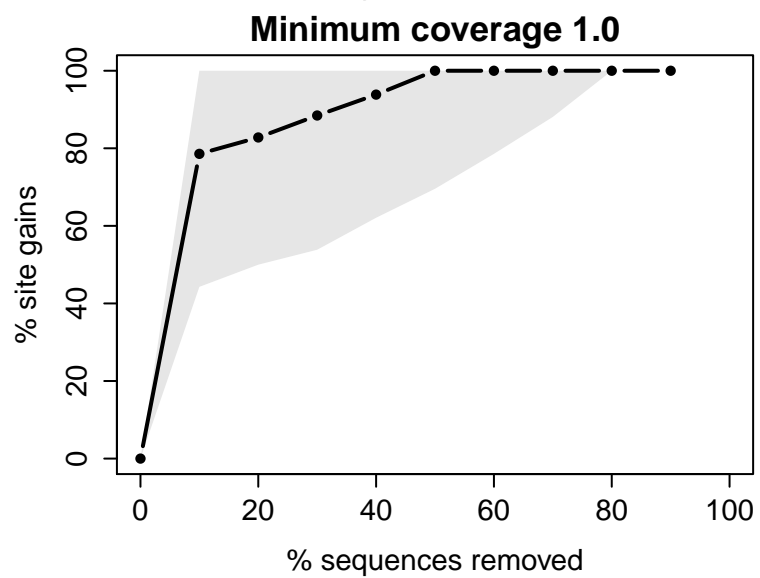

Supplement: Additional file 3 — Figure S2. Trade-off curves for the PFAM benchmark data set. Each panel represents a distinct procedure for generating the guide tree. The solid line shows the median over all 2,785 families; the shaded area represents the first (25 %) and third (75 %) quartiles. [file 12859_2015_619_MOESM3_ESM.pdf]
